# Calcium hydroxyapatite nanoparticles as a reinforcement filler in dental resin nanocomposite

**DOI:** 10.1007/s10856-021-06599-3

**Published:** 2021-10-03

**Authors:** Khalida Akhtar, Cynthia Pervez, Naila Zubair, Hina Khalid

**Affiliations:** Present Address: National Centre of Excellence in Physical Chemistry, University of Peshawar, Peshawar, 25120 Khyber Pakhtunkhwa Pakistan

## Abstract

The current study focuses on the fabrication of calcium hydroxyapatite (Ca_10_(PO_4_)_6_(OH)_2_) (HA) in a nanorange having whiskers- and cubic-shaped uniform particle morphology. The synthesized HA particles hold a promising feature as reinforcement fillers in dental acrylic resin composite. They increase the efficacy of reinforcement by length and aspect ratio, uniformity, and monodispersity. Therefore, the acrylic resin was reinforced with the as-synthesized monodispersed HA filler particles (0.2–1 Wt%). The presence of filler particles in the composite had a noticeable effect on the tribological and mechanical properties of the dental material. The morphological effect of HA particles on these properties was also investigated, revealing that cubic-shaped particles showed better results than whiskers. The as-fabricated composite (0.4 Wt%) of the cubic-shaped filler particles showed maximum hardness and improved antiwear/antifriction properties. Particle loading played its part in determining the optimum condition, whereas particle size also influenced the reinforcement efficiency. The current study revealed that particle morphology, particle size, uniformity, etc., of HA fillers, greatly influenced the tribological and mechanical properties of the acrylic resin-based nanocomposite. Improvement in the tribological properties of HA particle-reinforced acrylic resin composites (HA–acrylic resin) followed the trend as AR < C_m_C < WC < CC.

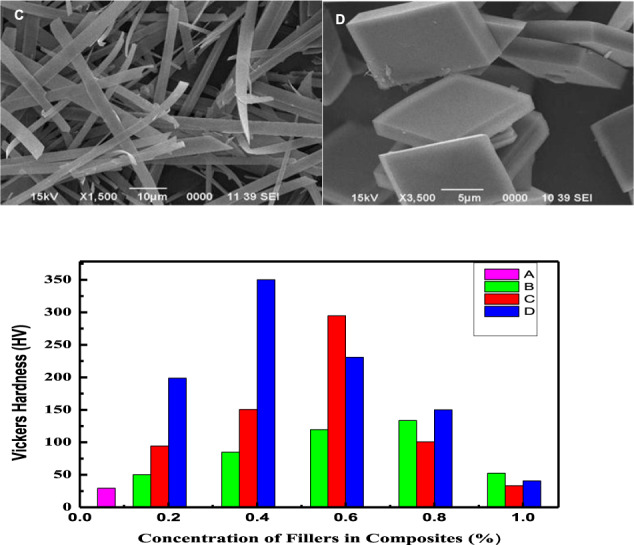

## Introduction

Nanoscale materials act as functional materials that possess exceptional and noticeable properties than bulk materials due to shape, size, geometry, morphology, and high surface-to-volume ratio [1–3]. These materials have many applications in various fields of catalysis, sensors, energy, and biomaterial production [4–6]. The active biomaterials are used as reinforcement fillers in the biocomposite to progress their properties [7]. Therefore, to achieve the desired properties, morphology controlled synthesis of uniform fine particles holds an essential role in nanoscience [5].

The concept of reinforcement fillers (biomaterials) in the composite polymer was first introduced by Bonfield in the 1980s [8]. Since then, biocomposites comprising of an organic polymeric matrix and inorganic reinforcement fillers have been massively synthesized. Among them, apatite and especially hydroxyapatite (HA) have gained considerable interest as reinforcement filler due to bioactivity and biocompatibility [9]. Calcium hydroxyapatite is generally known as HA. It forms bones and teeth major composition, so it is extensively used in orthopedics in bone repair and implants, as materials substitute in dentistry and as fillers in composites for medical applications [10, 11]. It is also used as a prime biomaterial because of its exceptional bioactive and osteoconductive features and biocompatibility with living tissue non-toxicity and bone attachment ability [12–14]. These applications require better adsorption capability, mechanical strength, and so on. To complement the mechanical properties of HA composite, few features need to be controlled like particle size, morphology, surface area, particle size distribution, agglomeration, structure, and crystallite size [14, 15].

HA can be fabricated by employing different synthesis routes, i.e., sol–gel, emulsion, hydrothermal, hydrolysis, etc. [16–19]. Still, the homogeneous precipitation method facilitates the particles with certain morphological features by controlling various reaction parameters [20, 21]. Also, this precipitation method is environmentally friendly and cost-effective [22].

Stable restorative synthetic dental materials with better bonding capabilities, which are physically, mechanically, and economically biocompatible with natural teeth and dentures, having high tensile strength, high-quality wear behavior, and ability to withstand high loads, have been a major concern among the researchers [23]. Furthermore, wear of teeth is a physiological process that includes erosion, the interplay of attrition and abrasion. Erosion is characterized by loss of tooth structure due to chemicals; the interplay of attrition, also known as two-body wear, is due to a tooth to tooth contact, whereas abrasion is caused by food motion over the teeth surface [24]. Wear on an enormous scale causes fatigue and decreased efficiency of masticatory muscles, loss of faulty tooth relationship, and vertical dimension of occlusion [25].

For the restoration of dental material and to overcome wear, resin composites and ceramics are massively used because of their enhanced properties [26]. But ceramics having a high elastic modulus show a mismatch in thermal expansion coefficient and are brittle [25]. Recently resins have eradicated ceramics due to lightweight, increased bonding abilities and lower fracture rate, but resins are more subjected to wear as compared to ceramics.

Nowadays, methacrylate-based resins are widely used for synthesizing artificial teeth and dentures. But the low wear rate of methacrylate-based resin remains a significant hindrance in its usage. So, the incorporation of inorganic fillers in the matrix is done to improve the wear properties of the resin composites [27]. Different types of inorganic fillers are used in resins, but among them, HA has shown immense benefits such as enhanced wear properties, better bonding ability both in the absence and presence of loads. In addition, it shows minute polymerization exotherm, improved mechanical properties, intrinsic opacity, and low shrinkage than the resin without HA fillers [28].

Moreover, the size of the inorganic fillers plays a vital role as well. Fillers in the nanorange enhance the properties of biocomposites by offering a high surface-to-volume ratio and better dispersion [9]. But still, HA particles showed some weaknesses, which cause a significant hindrance in the required performance of the resin composite regarding the mechanical strength and durability. Therefore, efforts have been made to synthesize HA fillers in a nanorange and with such morphology to overcome these drawbacks and provide better mechanical strength [29].

Moreover, researchers also carried out the cytotoxicity evaluation of HA particles and HA–PMMA composite by biocompatibility test and observed that HA and nontoxic cell cultures showed no significant difference. Hence, HA particles can be used as fillers in dental composites as they have no hazardous effect [30, 31].

Similarly, Motskin et al. [32] studied the particle load effect on toxicity and observed that a very high concentration of HA (>500 μg/ml) caused cell death, while low concentration exhibited no toxicity.

The reported literature showed that the mechanical and tribological properties of the composites reinforced with filler particles are dependent on sintering temperature, morphology, synthesis route, quality of the precursor material, and powder loading of the reinforced particles. Similarly, the interfacial bonding between the particles and matrix, homogenous dispersion, and the measurement method also plays a significant role in controlling the properties of the composites. These remarks made it clear that there is a need to synthesize monodispersed HA particles by establishing reproducible, and economical recipes and to check their performance as a filler in dental resin. Therefore, this study deals with the fabrication of monodispersed HA particles and HA-reinforced acrylic resin (AR)-based composites and evaluating the wear resistance and Vickers hardness of the as-synthesized HA–AR biocomposites.

## Experimental

### Materials

Analytical reagent grade anhydrous calcium nitrate (Ca(NO_3_)_2_, urea (NH_2_)_2_CO, sodium dihydrogen phosphate dihydrate (NaH_2_PO_4_.2H_2_O), NaOH, NaCl, KCl, CaCl_2_.2H_2_O, and Na_2_S.9H_2_O were purchased from BDH and Scharlau. Benzoyl peroxide (C_14_H_10_O_4_), Methyl-methacrylate-MMA (C_5_H_8_O_2_) and polymethyl-methacrylate-PMMA (C_5_H_8_O_2_)_n_ were purchased from Scharlau. All the stock solutions were filtered through a micropore membrane to remove the insoluble impurities.

### Synthesis of calcium hydroxyapatite

Calcium hydroxyapatite nanoparticles were prepared through homogenous hydrothermal precipitation by using calcium nitrate (0.01–0.5 M), sodium dihydrogen phosphate dihydrate (0.01–0.5 M), and urea (0.1M–0.5M). Stoichiometric amounts of the precursor solutions were mixed during optimization experiments [33]. The resulting mixture was then heated in a specially designed Pyrex glass tube at 70–90 °C in a water bath for predetermined time intervals (20–60 min). Furthermore, the resulting mixtures were kept under silent conditions during the reaction. Finally, reproducible recipes were established to synthesize uniform fine particles of calcium hydroxyapatite in whiskers and cubic shape. For whiskers, the reactant mixture containing 0.05 M Ca(NO_3_)_2_, 0.1 M NaH_2_PO_4,_ and 0.4 M urea was aged for 30 min at 85 °C, while the cubic morphology was precipitated when the same reactant mixture was aged for 50 min at the same temperature. Next, the dispersions were filtered through vacuum filtration, washed with ethanol and water. The particles obtained were then redispersed in 0.1 M NaOH and aged in the same medium for 30 min at 60 °C for conversion of monetite phase to HA. After this, the suspensions were filtered through a micropore filter and washed. The powder was then dried and stored for analysis and usage in the dental application.

The synthesized particles were characterized by SEM, FT-IR, X-ray diffraction (XRD), and TG/analysis techniques.

### Preparation of HA-reinforced acrylic resin-based nanocomposites

Before the preparation of the composite, dispersions of the selected as-synthesized HA particles having 0–1 Wt% were formed in 70 Wt% of MMA monomer with the aid of sonicator to ensure homogenous distribution of the particles. The obtained dispersion was further fused with 30 Wt% PMMA and 1.0% benzoyl peroxide, which acted as an initiator. Then, the mixture was stirred vigorously by a magnetic stirrer in specifically constructed 3 mm diameter Pyrex glass molds and permitted to stand overnight. Furthermore, the mixture was polymerized by heating at 37 °C and finally kept in an oven at 100 °C to ensure complete polymerization. As a result, various nanocomposites were synthesized, having different concentrations and morphology of HA filler particles. Moreover, commercial HA-based acrylic composite was also synthesized by following the same procedure with commercial HA and one without filler particles.

### Preparation of artificial saliva

Artificial saliva approximately 1 dm^3^ was prepared by composing an aqueous solution, having 0.4, 0.4, 0.795, 0.78, 0.005, and 1.0 gdm^–3^ of NaCl, KCl, CaCl_2_.2H_2_0, NaH_2_PO_4_.2H_2_0, Na_2_S.9H_2_O, and (NH_2_)_2_CO, respectively.

### Characterization of nanocomposites

The nanocomposites were analyzed by FT-IR (Shimadzu, IR Prestige-21, FTIR-8400S), to identify various organic (acrylic) and inorganic (HA) functional groups to ensure the complete reinforcement of HA into the acrylic matrix.

Similarly, the nanocomposites were further characterized by EDX (EDX Inca-200) for elemental analysis. For this purpose, the sample on the stub was pasted through double stick carbon tape, and the data were randomly collected from six different places and finally, an average value was taken into account. As a result, EDX analysis also ensured the complete reinforcement of HA into an acrylic composite.

### Properties of nanocomposites

The synthesized nanocomposites were tested to evaluate the following properties, and the effect of HA addition into the acrylic composite was monitored based on these properties.

#### Wear and friction measurement

Pin-on-disk tribometer was used to assess wear-resistance properties of acrylic nanocomposites with a diversified concentration of filler particles (HA) as well as commercially available AR composite. The wear resistance of each composite was measured in the presence and absence of artificial saliva. Before the experiments, the mild steel stubs were polished by silicon carbide paper having various grades (P-600, P-800, P-1200) using grinding machines (BenetecLabpol 8–12). Subsequently, the surface of the stub was activated and cleaned by sonicating them in NaOH (0.1 M) and HCl (0.01), respectively, for 5–10 min. For further cleaning, the stubs were washed with doubly distilled water and ethanol. The synthesized and the commercial AR composite were molded into pins of 3 mm diameter approximately. The pins were treated for wear measurement by fixing them in the upper static holder and rubbed against the rotating mild steel stub. The sliding distance (300 m) was kept the same throughout the experimentation. The speed of the motor was adjusted to 222 rpm. The readings were taken at maximum (8 N) and minimum load (2 N). The frictional force analysis was done with the help of a load transducer (Shimpo, Japan) connected to the computer, which continuously recorded the frictional force data with the aid of appropriate software. Frictional force data obtained was further used to calculate the friction coefficient. The density of pins was measured by densimeter before and after the experiment.

#### Vickers hardness

The Microhardness Tester (Shimadzu, HMV-2) was employed to determine Vicker’s hardness of the test composites. Before each analysis, the composite was indented with a pyramid diamond indenter having the applied load of 2 and 8 N. Measurement of two cross-diagonals was taken by machine build in scale. Each composite hardness was tested six times and the average value of Vicker’s hardness was accepted and marked as the final value.

## Results and discussion

### Synthesis of uniform fine particles of calcium hydroxyapatite

Calcium hydroxyapatite particle systems in uniform morphological features were prepared from the mixture containing urea, calcium nitrate Ca(NO_3_)_2_, and sodium dihydrogen phosphate NaH_2_PO_4_ in an appropriate amount at 80–95 °C in a different time period (20–50 min).

The formation of HA particles followed the following chemical reactions [21, 34]:

1 $${{{{{\mathrm{CO}}}}}}\left( {{{{{{\mathrm{NH}}}}}}_{{{{{\mathrm{2}}}}}}} \right)_{{{{{\mathrm{2}}}}}} + {{{{{\mathrm{H}}}}}}_{{{{{\mathrm{2}}}}}}{{{{{\mathrm{O}}}}}} \to {{{{{\mathrm{NH}}}}}}_{{{{{\mathrm{2}}}}}}{{{{{\mathrm{COONH}}}}}}_{{{{{\mathrm{4}}}}}}$$

2 $${{{{{\mathrm{NH}}}}}}_{{{{{\mathrm{2}}}}}}{{{{{\mathrm{COONH}}}}}}_{{{{{\mathrm{4}}}}}} \to {{{{{\mathrm{2NH}}}}}}_{{{{{\mathrm{3}}}}}} + {{{{{\mathrm{CO}}}}}}_{{{{{\mathrm{2}}}}}}$$

3 $${{{{{\mathrm{NH}}}}}}_{{{{{\mathrm{3}}}}}} + {{{{{\mathrm{H}}}}}}_{{{{{\mathrm{2}}}}}}{{{{{\mathrm{O}}}}}} \to {{{{{\mathrm{NH}}}}}}_{{{{{\mathrm{4}}}}}}^ + + {{{{{\mathrm{OH}}}}}}^ -$$

4 $${{{{{\mathrm{Ca}}}}}}\left( {{{{{{\mathrm{NO}}}}}}_{{{{{\mathrm{3}}}}}}} \right)_{{{{{\mathrm{2}}}}}} \to {{{{{\mathrm{Ca}}}}}}^{{{{{{\mathrm{2}}}}}} + } + {{{{{\mathrm{NO}}}}}}_{{{{{\mathrm{3}}}}}}^{{{{{{\mathrm{2}}}}}} - }$$

5 $${{{{{\mathrm{NaH}}}}}}_{{{{{\mathrm{2}}}}}}{{{{{\mathrm{PO}}}}}}_{{{{{\mathrm{4}}}}}} \to {{{{{\mathrm{Na}}}}}}^{{{{{{\mathrm{1}}}}}} + } + {{{{{\mathrm{H}}}}}}_{{{{{\mathrm{2}}}}}}{{{{{\mathrm{PO}}}}}}_{{{{{\mathrm{4}}}}}}^{{{{{{\mathrm{1}}}}}} - }$$

The products of reactions 3–5 (OH^1–^, Ca^2+^, H_2_PO_4_^1–^) led to the formation of CaHPO_4_ (monetite) (Eq. 6), which acts as a precursor in the formation of HA [35].

6 $${{{{{\mathrm{Ca}}}}}}^{{{{{{\mathrm{2}}}}}} + } + {{{{{\mathrm{H}}}}}}_{{{{{\mathrm{2}}}}}}{{{{{\mathrm{PO}}}}}}_{{{{{\mathrm{4}}}}}}^{{{{{{\mathrm{1}}}}}} - } + {{{{{\mathrm{OH}}}}}}^{{{{{{\mathrm{1}}}}}} - } \to {{{{{\mathrm{CaHPO}}}}}}_{{{{{\mathrm{4}}}}}} + {{{{{\mathrm{H}}}}}}^{{{{{{\mathrm{1}}}}}} + } + {{{{{\mathrm{H}}}}}}_{{{{{\mathrm{2}}}}}}{{{{{\mathrm{O}}}}}}$$

Monetite was further transferred to HA (Eq. 7) when immersed in aqueous NaOH [35, 36]:

7 $${{{{{\mathrm{10CaHPO}}}}}}_{{{{{\mathrm{4}}}}}} + {{{{{\mathrm{OH}}}}}}^{{{{{{\mathrm{1}}}}}} - } \to {{{{{\mathrm{Ca}}}}}}_{{{{{{\mathrm{10}}}}}}}\left( {{{{{{\mathrm{PO}}}}}}_{{{{{\mathrm{4}}}}}}} \right)_{{{{{\mathrm{6}}}}}}\left( {{{{{{\mathrm{OH}}}}}}} \right)_{{{{{\mathrm{2}}}}}} \downarrow + {{{{{\mathrm{4}}}}}}\left( {{{{{{\mathrm{PO}}}}}}_{{{{{\mathrm{4}}}}}}} \right)^{{{{{{\mathrm{3}}}}}} - } + {{{{{\mathrm{7H}}}}}}^ + + {{{{{\mathrm{H}}}}}}_{{{{{\mathrm{2}}}}}}{{{{{\mathrm{O}}}}}}$$

**Fig. 1:**
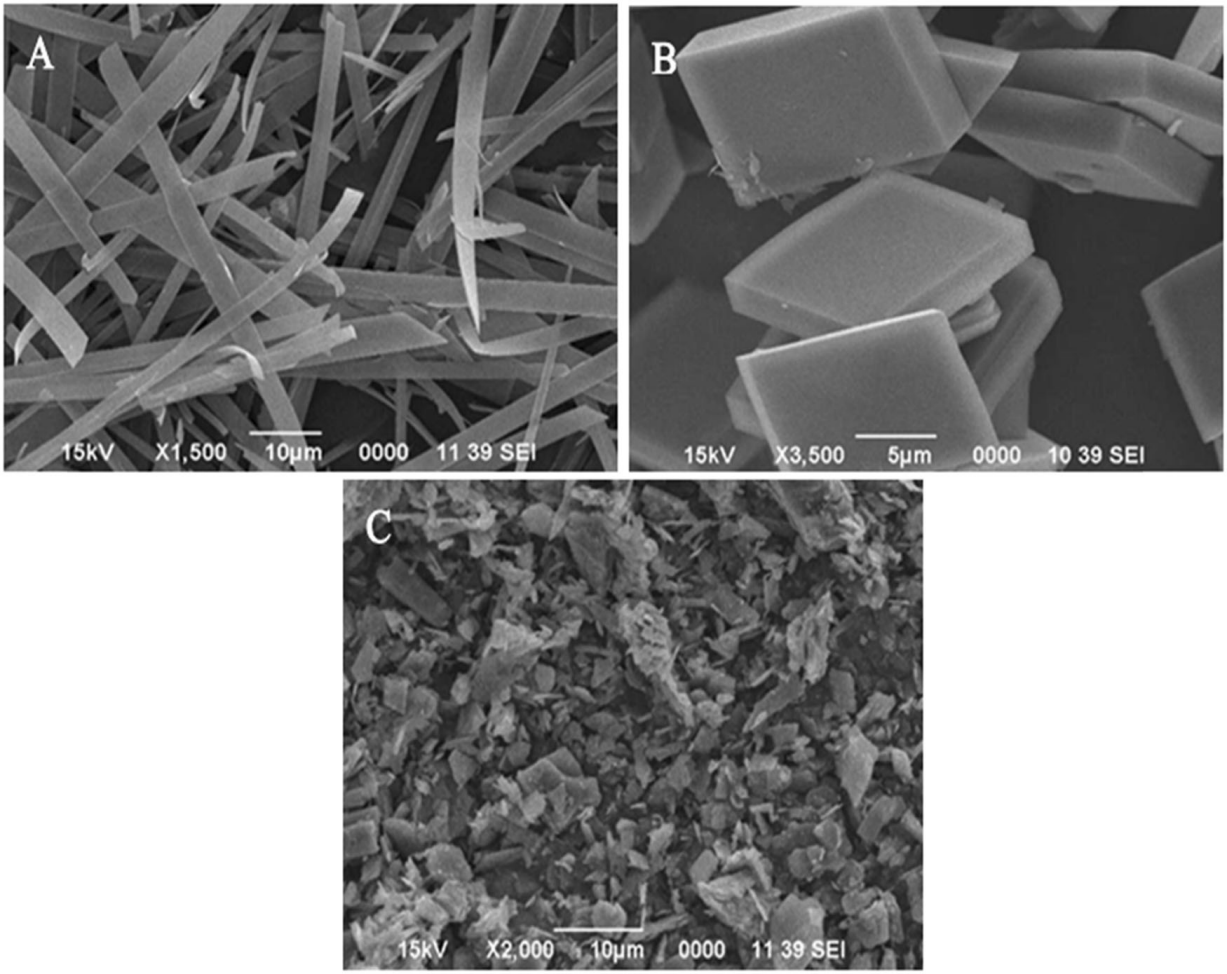
SEM analysis of the reactant solutions heated at 85 °C containing 0.4 M urea, 0.05 M Ca(NO_3_)_2_, and 0.1 M NaH_2_PO_4_ aged for (**A**) 30 and (**B**) 50 min, particles formed were immersed in 0.1 M NaOH solution for 30 min at 60 °C (**C**) commercial HA

The synthesized (HA) particle under various experimental conditions was analyzed through SEM micrographs. The observations revealed that reaction conditions played an important role in controlling the uniformity and monodispersity of the precipitated particle. It was also noted that the shape and size of the synthesized particles were significantly affected by the reaction conditions. Therefore, optimization of these parameters was required to explore the reproducible recipe for the required particle systems. By tailoring the technological conditions, i.e., reactant concentration, temperature, aging time, and pH, uniform HA particles in whiskers- (WHA, Fig. 1A) and cubic-shaped morphology (CHA, Fig. 1B) were produced after extensive optimization through a simple, environmentally friendly, and economical synthesis route.

### Characterization

The as-prepared uniform fine particles of HA designated as WHA (Fig. 1A) and CHA (Fig. 1B) were further analyzed by using different characterization techniques.

**Fig. 2:**
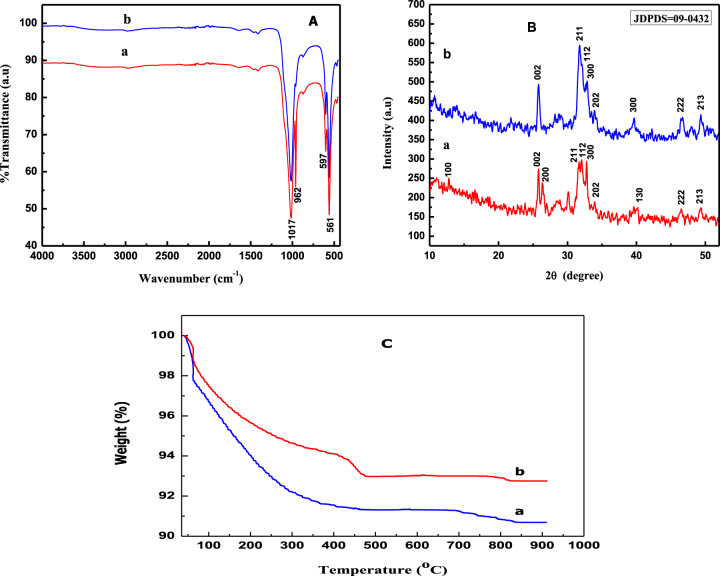
**A** FT-IR spectra, **B** XRD spectra, and **C** TGA curves of WHA (**a**) and CHA (**b**) particles

The FT-IR spectra of the as-synthesized monodispersed (whiskers (WHA) and cubic (CHA)) are shown in Fig. 2A. The characteristic band of the crystalline HA was observed at 1017 cm^−1^ for whiskers (Fig. 2Aa) and cubic (Fig. 2Ab) particles that authenticated the presence of P-O-H [23].

Similarly, the IR spectra portrayed the characteristic stretching vibration of PO_4_^–3^ at 561 and 597 cm^−1^ in both samples [37, 38]. Besides, the bending vibration at 962 cm^–1^ of PO_4_^–3^ was well shown in WHA (Fig. 2Aa), whereas the same band was diminished in HA particles having cubic morphology (Fig. 2Ab) [39].

The selected HA particles (WHA and CHA) shown in Fig. 1A, B were further examined for the crystallinity, phase purity, and composition by the XRD, as shown in Fig. 2Ba, b respectively. The observed peaks were well coordinated with card no. ICDD (09-0432) and the spectra identified the analyzed material as HA having hexagonal phase with lattice parameters of *a* = *b* = 0.9407 nm and *c* = 0.6873 nm [40, 41].

Thermal gravimetric analysis in the temperature range of 40–1000 °C (Fig. 2C) presented 9.4% and 7.3% of weight loss, respectively, for WHA (Fig. 2Ca) and CHA (Fig. 2Cb), which were close to the values calculated from the proposed thermal initiated reactions (Eqs. 8–9).

8 $${{{{{\mathrm{Ca}}}}}}_{{{{{{\mathrm{10}}}}}}}\left( {{{{{{\mathrm{PO}}}}}}_{{{{{\mathrm{4}}}}}}} \right)_{{{{{\mathrm{6}}}}}}\left( {{{{{{\mathrm{OH}}}}}}} \right)_{{{{{\mathrm{2}}}}}}{{{{{\mathrm{.3/2H}}}}}}_{{{{{\mathrm{2}}}}}}{{{{{\mathrm{O}}}}}}\mathop{\longrightarrow}\limits^{{{{{\mathrm{wt}}}}\,{{{\mathrm{loss}}}} = 9.8\% }}{{{{{\mathrm{3Ca}}}}}}_{{{{{\mathrm{3}}}}}}\left( {{{{{{\mathrm{PO}}}}}}_{{{{{\mathrm{4}}}}}}} \right)_{{{{{\mathrm{2}}}}}} + {{{{{\mathrm{CaO}}}}}} + {{{{{\mathrm{5/2H}}}}}}_{{{{{\mathrm{2}}}}}}{{{{{\mathrm{O}}}}}}$$

9 $${{{{{\mathrm{Ca}}}}}}_{{{{{{\mathrm{10}}}}}}}\left( {{{{{{\mathrm{PO}}}}}}_{{{{{\mathrm{4}}}}}}} \right)_{{{{{\mathrm{6}}}}}}\left( {{{{{{\mathrm{OH}}}}}}} \right)_{{{{{\mathrm{2}}}}}}{{{{{\mathrm{.1/5H}}}}}}_{{{{{\mathrm{2}}}}}}{{{{{\mathrm{O}}}}}}\mathop{\longrightarrow}\limits^{{{{{\mathrm{weight}}}}\,{{{\mathrm{loss}}}} = 7.5\% }}{{{{{\mathrm{3Ca}}}}}}_{{{{{\mathrm{3}}}}}}\left( {{{{{{\mathrm{PO}}}}}}_{{{{{\mathrm{4}}}}}}} \right)_{{{{{\mathrm{2}}}}}} + {{{{{\mathrm{CaO}}}}}} + {{{{{\mathrm{6/5H}}}}}}_{{{{{\mathrm{2}}}}}}{{{{{\mathrm{O}}}}}}$$

The observed difference in the weight loss was due to the variance in the morphology of the particles as it has been testified elsewhere that whisker-shaped particles have the ability to retained high water content in comparison to cubic-shaped structures [42].

The synthesized particles (WHA, CHA) were analyzed for specific surface area and it was observed that the cubic particles showed high surface area (137.461 m^2^/g) in comparison to the whisker-shaped (104.485 m^2^/g) particle morphology. These values showed the dependence of the surface area of HA particles with their particle morphology [43, 44].

### Fabrication of nanocomposites

After the complete characterization, the HA nanoparticles synthesized by homogeneous precipitation shown in Fig. 1A, B were used as reinforcement fillers in acrylic-based nanocomposites. The composites having a variable concentration of reinforced HA were characterized by FT-IR and EDX to ensure the complete dispersion of HA into acrylic composites. HA-reinforced nanocomposites were further subjected to tribological and mechanical tests and their wear resistance, frictional coefficient, and hardness properties were evaluated.

The AR having no HA fillers was designated as AR, whereas the composite containing the synthesized HA fillers was termed as a hybrid composite represented by WC (whisker composite) and CC (cubes composite). The commercial HA particle-reinforced resin composite was termed as CmC. The composites were characterized by the following techniques before the tribological and mechanical tests to ensure that HA fillers have been dispersed homogeneously in the blank matrix.

#### FT-IR analysis of the composites

**Fig. 3:**
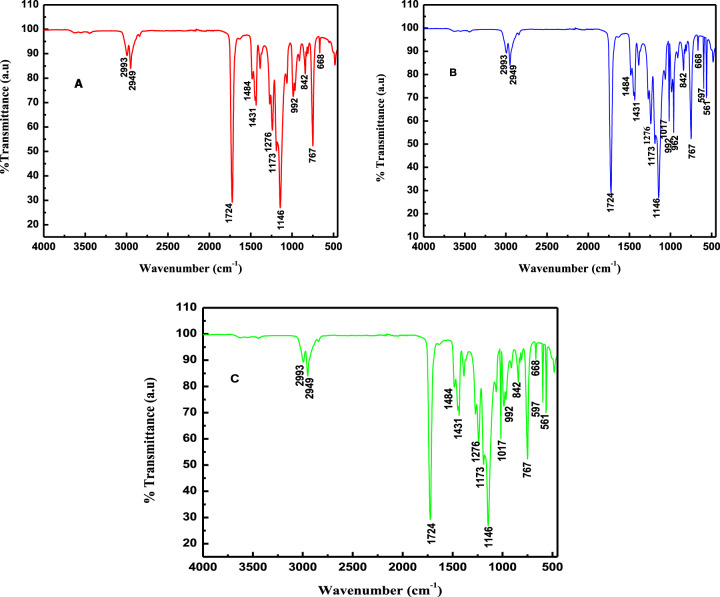
FT-IR spectra of AR (**A**) and HA-reinforced nanocomposites, WC (**B**) and CC (**C**)

The FT-IR spectrum of blank AR comprised of PMMA polymer and MMA monomer is shown in Fig. 3A. It consists of the sharp characteristic band at 749, 1173, and 1724 cm^−1^, corresponding to the stretching vibration of ester carbonyl group C=O [45–47], whereas the distinctive bands assigned to the presence of ester stretching bond C-O was located at 1146, 1276, and 1483 cm^−1^ [45, 46]. Similarly, the weak bands originated at 2993 and 2949 cm^−1^ were attributed to the stretching vibration of the C-H bond. The band centered at 668 and 1431 cm^−1^ were due to the bending and stretching vibration of CH_2_ and CH_3_ groups, respectively [45, 46]. The tension and stretching bands of C-C gave a sharp peak at 840 and 992 cm^−1^ [48, 49]. Furthermore, no band was observed at 1630 cm^−1^, corresponding to C=C, which revealed the complete polymerization of MMA into PMMA [49].

Similarly, the FT-IR spectra of WC and CC are shown in Fig. 3B and Fig. 3C, respectively. Spectra showed all the essential peaks of the matrix (Fig. 3A) and that of HA (FT-IR, Fig. 2A) (Table 1), thus demonstrating that HA was successfully reinforced in the composites. HA peaks included the characteristic peak at 1017 cm^−1^ corresponding to P-O-H. Moreover, PO_4_^–3^ stretching band was centered at 561 and 597 cm^−1^, while the bending vibration of PO_4_^–3^ at 962 cm^−1^ appeared only in WC.

**Table 1:** Wavenumber position and IR absorption of different chemical groups on solid surfaces shown in Fig. 3

Band position (cm^−1^)			Group specie	Mode of vibration	References
AR (Fig. 3A)	WC (Fig. 3B)	CC (Fig. 3C)			
767, 1173, 1724	767, 1173, 1724	767, 1173, 1724	C=O	Stretching	[44–46]
1146, 1276, 1484	1146, 1276, 1484	1146, 1276, 1484	C-O	Stretching	[44, 45]
2993, 2949	2993, 2949	2993, 2949	CH	Stretching	[44]
842, 992	842, 992	842, 992	C-C	Stretching	[47, 48]
1431, 668	–	–	CH_2_ and CH_3_	Stretching and bending	[44, 45]
–	1017	1017	P-O-H	Bending	[23]
–	962	–	PO_4_^–3^	Bending	[38]
–	597	597	PO_4_^–3^	Stretching	[37]
–	561	561	PO_4_^–3^	Stretching	[36]

#### EDX analysis of the composites

**Fig. 4:**
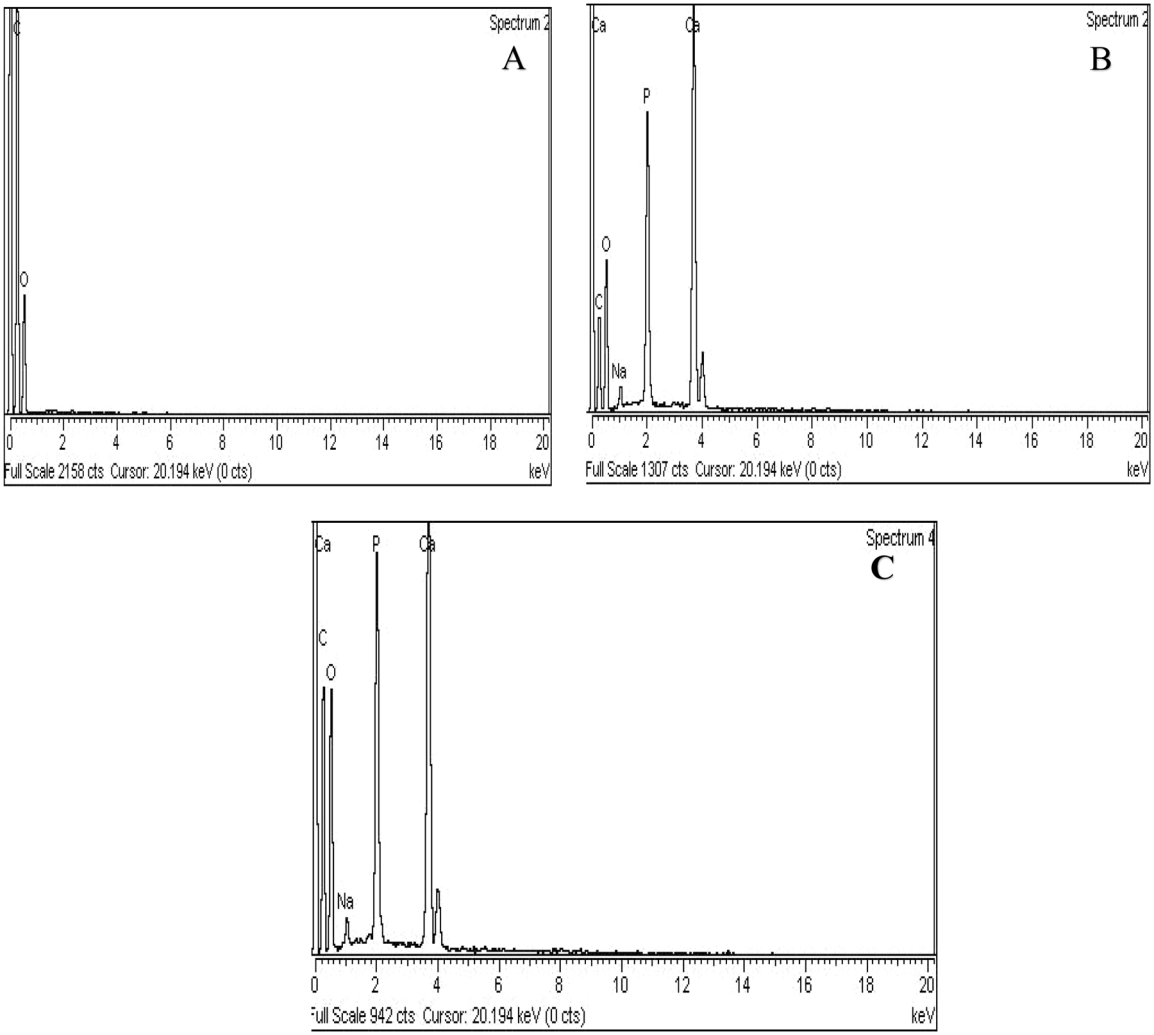
EDX spectra of AR (**A**) and hybrid nanocomposites (**B**) WC and (**C**) CC

The metal contents of the blank matrix (AR) and composites (WC and CC) were analyzed quantitatively by EDX (Fig. 4 and Table 2). The spectrum of AR (Fig. 4A) demonstrated the sharp, clear peaks of C and O in the matrix in different percentages. Moreover, the EDX spectra of WC and CC composite (Fig. 4B, C) contained various elements, i. e., C, O, Ca, H, P, and Na. The given spectra also revealed that Na residue was leached out during washing and was present in the trace amount in the composites, as reported in the literature [50]. The remaining peaks of C and O corresponded to AR, while Ca, H, and P designated the presence of HA particles in the composites [51].

**Table 2:** EDX analyzed elements of AR, WC, and CC

Elements	Weight%			Atomic%		
	AR (Fig. 4A)	WC (Fig. 4B)	CC (Fig. 4C)	AR	WC	CC
Carbon	56.50	32.69	34.12	63.37	45.57	46.48
Oxygen	43.50	39.78	40.77	36.63	41.83	41.18
Calcium		17.89	15.33		7.28	4.68
Phosphorous		9.03	8.83		4.88	6.98
Sodium		0.61	0.95		0.44	0.68
Total	100	100	100	100	100	100

### Antiwear and antifriction resistance of the composites

The matrix (AR) and hybrid nanocomposites (WC, CC) were subjected to wear and friction tests. For comparison purposes, a composite was synthesized by reinforcing commercial HA particles of irregular shape and size (Fig. 1C) in the polymer matrix and was designated as C_m_C (Commercial composite). The pin-on-disk tribometer was used for evaluating the wear-resistance properties of the test composites. The main focus of the study was to synthesize an economically feasible hybrid composite having better tribological properties and, secondly, to study the effect of particle morphology and uniformity of the reinforced HA particles on the tribological properties of the composites. Therefore, a narrow set of experimental conditions were employed for the wear-resistance and coefficient of friction (COF) measurement, as shown in Table 3. Moreover, tribological tests were conducted in the dry (in the absence of artificial saliva) and wet (in artificial saliva) conditions. The artificial saliva was synthesized by using various salts in different proportions [52].

**Table 3:** Optimum experimental parameters applied for wear-resistance measurement

S. no.	Experimental parameter	Optimized value
1	Sliding distance	300 m
2	Time	15 min
3	Speed	222 rpm
4	Temperature	25 °C
5	Minimum load	2 N
6	Maximum load	8 N

**Fig. 5:**
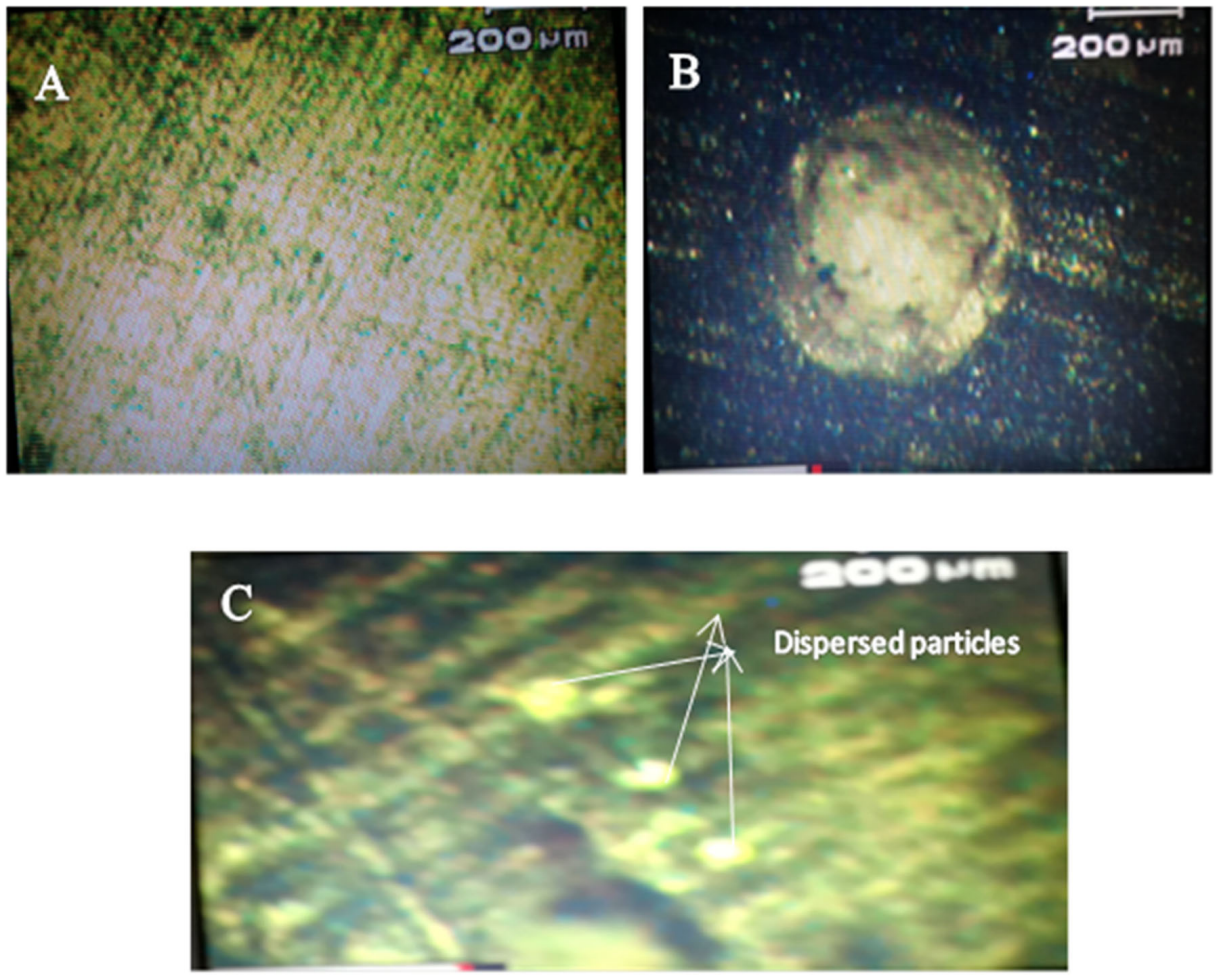
Microscopic images of **A** AR, **B** C_m_C, and **C** hybrid composite

Furthermore, HA filler particles were added in multiple concentrations, i. e., 0.2–1 Wt% in the MMA monomer. Finally, the mixture was sonicated for 5 h to ensure homogeneous dispersion of HA fillers in the matrix. The AR, C_m_C, and the hybrid composites (WC and CC) were then analyzed using a compound microscope (Fig. 5). As can be seen from the images (Fig. 5A), no particles were observed in AR.

The C_m_C showed the dispersion of particles, but slight agglomeration occurred in the matrix, as displayed in Fig. 5B. In contrast, the hybrid composite, as shown in Fig. 5C, revealed the homogenous dispersion of particles and they remained intact due to the homogeneity and uniformity achieved during their synthesis [53].

The wear rate of different composites at a minimum (2 N) and maximum (8 N) applied load was calculated by taking % weight loss of the pins when it rubbed against the mild steel stubs under a given load and sliding distance at room temperature (Eq. 10). The wear rate of various composites (AR, C_m_C, WC, CC) was plotted against the Wt% concentration of fillers under two extreme loads (minimum 2 N and maximum 8 N) in dry and wet conditions.

10 $${{W}} = \delta {{m}}/{{LDF}}$$

Where *δm* is the weight loss (%), *W* the wear rate (mm^3^/Nm), *L* the sliding distance (m), *D* the density (g/mm^3^) (the density of the pins was measured by densimeter), and *F* is the force (N).

Moreover, the COF at the point of sliding contact was calculated from the friction data recorded by a load transducer using Eq. 11:

11 $$\mu = {{F}}/{{N}}$$

where *µ* is the friction coefficient, *F* the frictional force, and *N* is the applied load vertical to the axis of rotation.

### Optimization for the fabrication of nanocomposite

The effect of different parameters such as concentration, morphology, and particle size on frictional and wear-resistance properties of various composites were studied to explore the experimental parameters for the synthesis of the optimized blend of HA filler particles in the polymer matrix.

#### Effect of fillers concentration

**Fig. 6:**
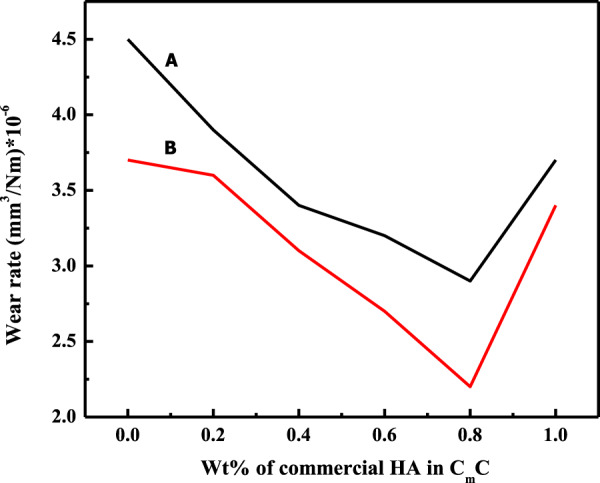
Wear rate of C_m_C during wear experiment conducted at 2 N applied load and 300 m sliding distance at room temperature in **A** dry and **B** wet condition

**Fig. 7:**
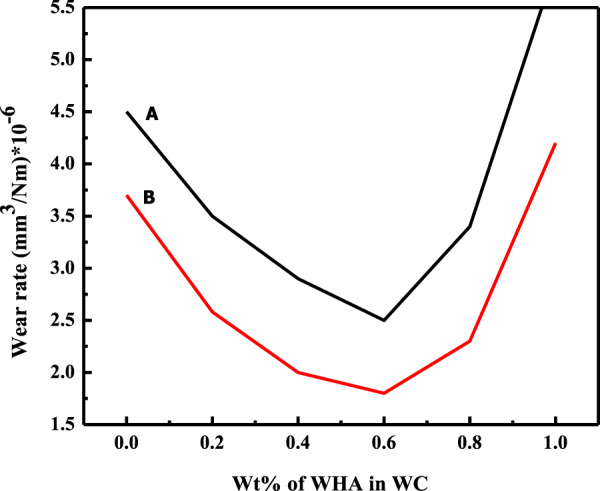
Wear rate vs. percent amount of the filler in WC **A** dry and **B** wet condition during wear experiment conducted at 2 N applied load and 300 m sliding distance at room temperature

**Fig. 8:**
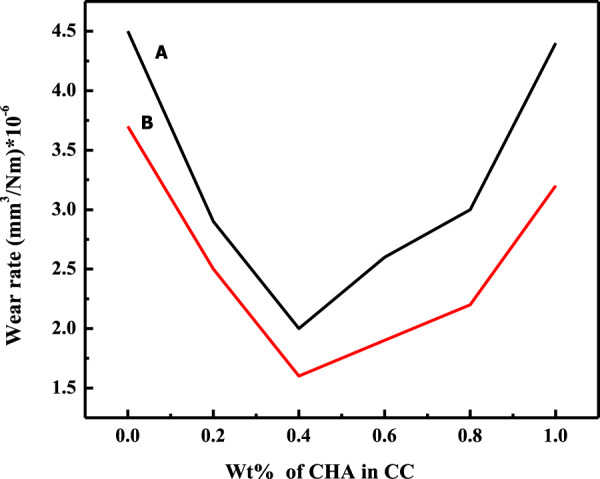
Wear rate of CC during wear experiment conducted at 2 N applied load and 300 m sliding distance at room temperature in **A** dry and **B** wet condition

The concentration of filler particles in the composites played a key role in enhancing the tribological properties. The effects of fillers concentration on the wear rate of various composites (C_m_C, WC, CC) under 2 N (minimum) applied load in dry and wet conditions are shown in Figs. 6–8, respectively. These figures revealed that the wear rate decreased with the increase in Wt% of the filler particle in the composite to a certain limit (optimum limit), after which it increased. As for the C_m_C (Fig. 6), the addition of filler particles in the matrix resulted in a gradual decrease in the wear rate up to the 0.8 Wt% concentration of the filler, after which an abrupt increase was observed at 1 Wt%. It showed that 0.8 Wt% of the particles were the optimum concentration at which the minimum % weight loss and minimum wear rate were observed in the C_m_C in both dry and wet conditions. At optimum concentration, i. e., 0.8 W%, the wear rate was reduced by 35.6% in dry and 40.6% in wet conditions as compared to the wear rate of the blank matrix (AR). As can be seen, the composite material loss in the wet condition was less as compared to the loss in a dry condition, which showed a lubricating behavior of the artificial saliva. Moreover, the wear rate of the WC and CC, as portrayed in Figs. 7 and 8, followed the same trend, but the observed optimum limit of the WHA and CHA particles was 0.6 and 0.4 Wt% in WC and CC, respectively. In this case, the calculated decrease in the wear rate for the AR was 44.4% and 51.35% for WC, whereas CC showed a 55.55% and 56.8% decrease in a wear rate, respectively, in dry and wet conditions. The percent decrease in the wear rate of the composites concerning its matrix varies from system to system depending upon the particle morphology of the fillers and other experimental conditions. A 5.5% and 35.2% decrease in the wear rate was observed at 2 and 8 N, respectively, for C_m_C for the PMMA matrix in a dry condition [54].

These observations pointed to the fact that the addition of filler particles improved the wear-resistance property of the polymer matrix [54]. Furthermore, the particle uniformity and particle morphology of the added particles strongly affected the optimum concentration of the particles in the composite as high concentration (0.8 Wt%) of the commercial irregular and nonuniform particles (Fig. 1C) was required compared to the concentration of uniform monodispersed whiskers (0.6 Wt%) and cubic (0.4 Wt%) shaped particles for the decrease in wear rate among their respective set of data (Figs. 6–8). In response to the particle morphology of the filler particles in the present case, the cubic-shaped HA particles showed an excellent response to the wear resistance.

**Fig. 9:**
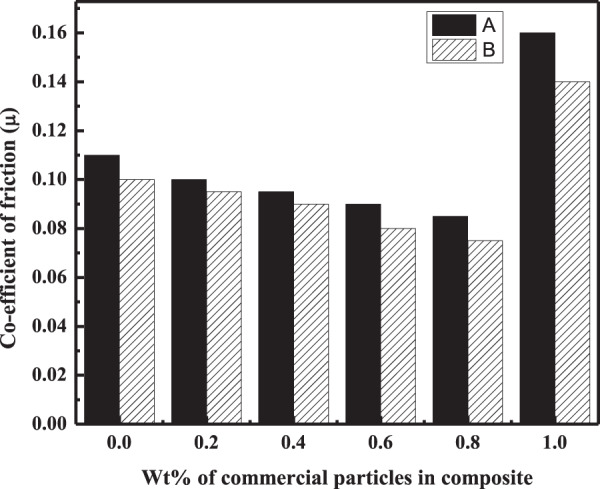
Coefficient of friction of C_m_C during wear experiment conducted at 2 N applied load and 300 m sliding distance at room temperature in **A** dry and **B** wet condition

**Fig. 10:**
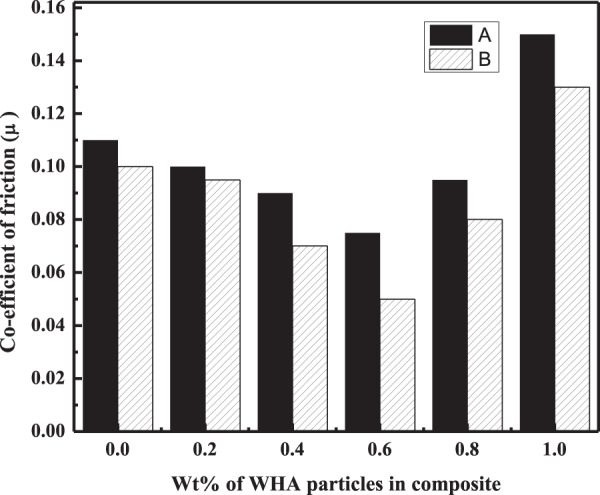
Coefficient of friction of WC vs. Wt% concentration of the filler in the composite conducted at 2 N applied load and 300 m sliding distance at room temperature in **A** dry and **B** wet conditions

**Fig. 11:**
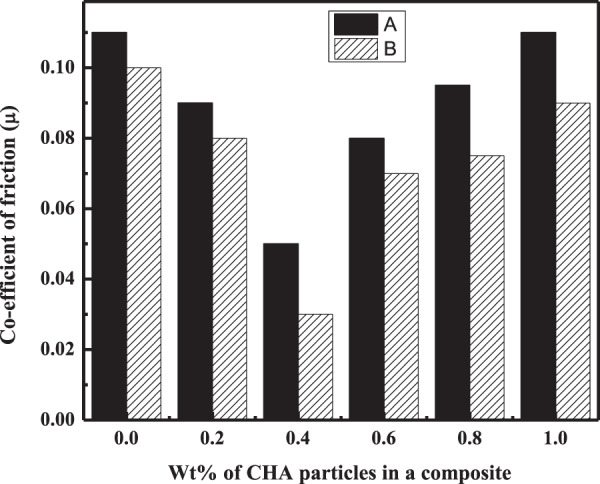
Coefficient of friction of CC vs. Wt% concentration of the filler in the composite conducted at 2 N applied load and 300 m sliding distance at room temperature in **A** dry and **B** wet condition

In addition to the wear rate, variation in the COF of composites in dry and wet conditions was also calculated from Eq. 11 and plotted against Wt% of the filler particles in the polymer matrix. It was noted for all the three composites that the friction coefficient values were in agreement with the results of the wear rate. As shown in Figs. 9–11, the minimum COF was observed for the composites having 0.8, 0.6, and 0.4 Wt% of the filler for C_m_C, WC, and CC, respectively, in dry and wet condition. The percent decrease in the friction coefficient of the HA–AR composites concerning the filler amount from the blank (AR) in the dry and wet environment is listed in Table 4. As can be seen, the percent decrease in the COF of C_m_C was less compared to the WC and CC under the same experimental conditions. The percent decrease in the COF of the composites concerning its blank varies from system to system depending upon the chemical nature and morphological features of the fillers particles and its composition in the polymer matrix [55].

**Table 4:** Percent decrease in the wear rate and friction coefficient of the composites C_m_C, WC, and CC having an optimum concentration of the filler particles with respect to AR

Nanocomposite codes	Decrease in wear rate (%)				Decrease in friction coefficient (%)			
	Applied load (2 N)		Applied load (8 N)		Applied load (2 N)		Applied load (8 N)	
	Dry condition	Wet condition	Dry condition	Wet condition	Dry condition	Wet condition	Dry condition	Wet condition
C_m_C	35.50	40.54	33.30	37.50	22.73	25.07	21.4	22.18
WC	44.19	50.95	45.80	55.00	31.90	50.20	35.7	36.30
CC	55.55	56.75	50.0	69.23	54.40	34.70	39.3	45.40

Moreover, the same tribological properties (wear rate and COF) of the fabricated composites having different Wt% of the filler particles were tested at a maximum load of 8 N, while other experimental parameters were the same. The results (Tables 5 and 6) of these properties for the fabricated three HA–AR (CmC, WC, CC) composite systems followed the same trend as was observed under the applied load of 2 N (Figs. 6–8). In this case, too, the lowest wear rate and COF were found for the same composites as were observed for the applied load of 2 N. The percent decrease in the wear rate and COF of the optimum composites for AR are presented in Table 4.

**Table 5:** Wear rate of C_m_C, WC, and CC during wear experiment conducted at 8 N applied load and 300 m sliding distance at room temperature in dry and wet condition

Conc of fillers (Wt %)	Wear rate (mm^3^/Nm)×10^–6^					
	C_m_C		WC		CC	
	Dry condition	Wet condition	Dry condition	Wet condition	Dry condition	Wet condition
0	4.8	3.9	4.8	3.9	4.8	3.9
0.2	4	3.5	3.7	2.9	3.1	2.7
0.4	3.6	3.2	2.9	2	**2.4**	**1.2**
0.6	3.33	3.1	**2.6**	**1.8**	2.9	2.5
0.8	**3.2**	**2.5**	3.7	2.5	3.4	3
1.0	4.5	3.9	6.2	5.6	4.7	3.8

Bold entries represent optimum properties

**Table 6:** COF of C_m_C, WC, and CC during wear experiment conducted at 8 N applied load and 300 m sliding distance at room temperature in dry and wet condition

Conc of fillers (Wt %)	Coefficient of friction (COF) (µ)					
	C_m_C		WC		CC	
	Dry condition	Wet condition	Dry condition	Wet condition	Dry condition	Wet condition
0	0.14	0.11	0.14	0.11	0.14	0.11
0.2	0.133	0.11	0.13	0.12	0.10	0.08
0.4	0.131	0.095	0.1	0.09	**0.085**	**0.06**
0.6	0.12	0.09	**0.09**	**0.07**	0.093	0.072
0.8	**0.11**	**0.085**	0.11	0.095	0.09	0.08
1.0	0.19	0.18	0.17	0.15	0.13	0.10

Bold entries represent optimum properties

As can be seen from the antiwear and anti-frictional data at a minimum (2 N) and maximum load (8 N) (Table 4) of the optimum HA–AR composites, addition of the filler, irrespective of the particle uniformity and morphology, improved the tribological properties of the polymer matrix. This can be attributed to the fact that grooves were present at the sliding contact. The addition of the filler particles enabled them to get accumulated in the grooves, thus flattening the contact area and providing extra protection against wear [55]. It was further seen that the filler particles remained productive in the wear reduction until an optimum concentration by forming a tribofilm layer. This layer played an influential role in the reduction of wear. But the further increase in filler particles weakened the bond between the tribofilm layer and the test stubs. Also, an increase in the number of filler particles also caused collision among themselves; thus, fewer particles were deposited at the grooves; therefore, wear resistance decreased after an optimum concentration [50].

Secondly, the composite with the monodispersed HA filler particles (WC and CC) showed maximum resistance against the wear and friction compared to the action of the C_m_C. It demonstrated that particle uniformity had a significant effect on the strengthening of the matrix [56].

#### Effect of fillers particle morphology

The size and shape of the filler nanoparticles played a significant role in determining the tribological properties of the nanocomposites [57]. It greatly affected the frictional behavior as well as the antiwear properties to some extent. In the present study, it is evident from the antiwear and anti-frictional data (Figs. 6–11) that C_m_C having the incorporated large-sized irregular particles (Fig. 1C) caused insignificant improvement in the tribological properties (Table 4) as compared to WC and CC comprised of monodispersed uniform fine particles in the polymer matrix. It clearly demonstrated that the synthesized small-sized monodispersed particles of HA (WHA and CHA) were uniformly distributed in the matrix during the composite formation and strengthen the composite, which resisted more against the friction and wear as compared to AR and C_m_C. As the hybrid composites comprised of fillers particles in the nanometer range, their small particle size enabled them to penetrate easily at the point of sliding contact [58], thus reduced COF values and caused lubrication.

Furthermore, their high surface-to-volume ratio provided ease of reacting with the environment. In addition, an inspection of Table 4 portrayed that the particle morphology of the additives also played an essential role in the antiwear and antifriction performance of the formed composite. As in the present case, CC showed better performance than WC due to the difference in the shape of the incorporated HA filler particles (WHA-SEM Fig. 1A and CHA-SEM Fig. 1B). In addition, the homogenous dispersion of the particles caused the feeding effect in which particles of different sizes behaved similarly, thus enhanced the properties [49].

The difference in the performance of the two optimum composites (WC and CC) having the as-synthesized HA particles of whisker and cubic morphology (WHA and CHA) can be explained based on the interaction of the incorporated particles and the contacting layers. Accordingly, the synergism between the adjacent layers played an important role in determining the frictional behavior [59]. Furthermore, HA particles exhibited weak Vander Waal forces and they were extended for just a few nanometers; therefore, CC and WC followed the exfoliation mechanism [56, 60]. In this mechanism, under the effect of shear force, two adjacent layers were easily exfoliated, which led to the sliding movement of these layers, followed by a decrease in frictional and wear value.

The CC composite possessed an additional interaction besides the exfoliation mechanism [57]. This interaction formed the relation between the outer layer and the substrate, which linked the surface energy of the basal plane to the property of the environment. The number of layers and their interspacing also played a key role and caused a significant effect on the tribological properties of the composites. CC composites possessed low interspacing and several layers, which aided in improving the friction and wear property of the cubic particles [59].

### Vickers hardness of the composites

**Fig. 12:**
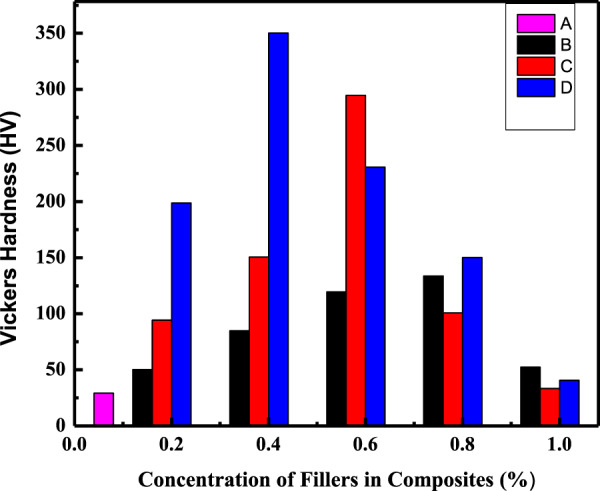
Variation of Vicker’s hardness as a function of filler concentration in the composites AR (**A**), C_m_C (**B**), WC (**C**), and CC (**D**) at 2 N load

**Fig. 13:**
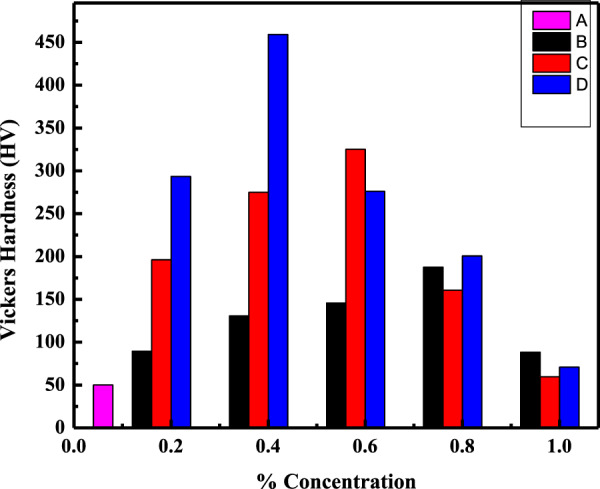
Vicker’s hardness of AR (**A**), C_m_C (**B**), WC (**C**), and CC (**D**) against % concentration of fillers in these composites at 8 N

The AR- and the HA-reinforced AR composite with different filler particles’ concentrations were further evaluated for their mechanical properties. Vicker’s hardness was measured by using the micro-indentation technique for 5 s by applying 2 and 8 N load (Figs. 12 and 13). The microhardness value for the AR was comparable to the reported literature at both loads [61]. The main focus of the study was to achieve an optimum level of the filler concentration at which both materials interact effectively, thereby increasing the tensile strength of the composite and hence acquired the enhanced mechanical properties. The study of tribological properties revealed that at a particular optimum incorporated concentration (0.8 Wt% for commercial, 0.6 Wt% for WHA, and 0.4 Wt% for CHA) of the HA filler particles, the composite experienced minimum wear rate and COF. Hence the test composites were studied to check the improved interaction between the particles and AR matrix in the form of hardness, which was then correlated with the wear and friction data explained previously.

Vicker’s hardness measured at two different loads was plotted against the composites with different % concentration (0–1 Wt%) of the filler particles, as illustrated in Figs. 12 and 13. As can be seen, in both cases, Vicker’s hardness increased with the addition of filler particles in the composite systems up to a certain limit, whereas further addition of the filler in the polymer matrix decreased Vicker’s hardness of the composite. It may be due to the fact that the added particles worked as a deformation lock both in the extensional and shear mode; thus, increase in the filler particle concentration increased Vicker’s hardness of the CmC, WC, and CC, having the incorporated HA particles up to 0.8, 0.6 and 0.4 Wt%, respectively. These results strongly supported the results obtained during the wear and friction study. Thus, the composite achieved an optimum level of interfacial stress at this concentration due to enhanced mechanical interlocking.

Further increase of the concentration decreased Vicker’s hardness of the composite due to high particle loading. It led to the formation of voids and cavities, which served as a nucleation site and promoted the link-up modes and internal crack tips of the filler particles [48, 61]. This caused the stress overload of the remaining polymer matrix and, finally, wearing started, which destroyed the whole structure of the composite. Secondly, at a high concentration of the filler particles, agglomeration occurred due to weak Vander Waal forces, thereby reducing the interaction, resulting in the decreased mechanical properties of the composites [60]. Moreover, in the PMMA matrix, a high concentration of the filler particles caused less PMMA matrix to intercalate into HA distribution, thus weakening the bond between them and finally decreased the hardness of the composite [53, 62]. The increase in hardness of the AR due to the reinforcement of the added particles for the blank matrix (AR) followed the trend as AR < C_m_C < WC < CC (Table 7). The microhardness value for AR was comparable to that reported in the literature [61]. The lower value of C_m_C as compared to both the hybrid composites was due to the lower tensile strength. It was weakened by the cohesive strength of the agglomerated filler particles in the matrix, as shown in Fig. 5B, which further reduced the bond strength of the HA–PMMA interface. Therefore, the limiting factor in CmC was the reduced interfacial bonding [53]. The increased hardness of the hybrid composites corresponded to the higher bond strength of the HA–PMMA interface as there were no cohesive forces and the particles were homogeneously distributed and remained intact, as displayed in Fig. 5C. Among the hybrid composites, the CC showed higher hardness as compared to WC as reported before [63], which can be attributed to the fact that condensation of water vapors can easily occur between whiskers forming a chemical bond that promoted the agglomeration of whiskers in WC as compared to the cubes in CC [43]. The obtained data were compared with the hardness results of other research groups [61, 63–65] and the tabulated data (Table 8) showed that improvement in hardness of the teeth material was either a result of high concentration of the fillers or due to sintering of the composite at high temperatures [63–65], while in the present case, addition of only 0.6 and 0.4 Wt% of the as-synthesized uniform whiskers (WHA) and cubic (CHA) shaped particles of HA increased the hardness of the acrylic dental resin up to 458 and 324 HV, which were respectively 12 and 10 times greater than its matrix hardness.

**Table 7:** Increase in Vicker’s hardness of the HA–AR optimum composites with respect to the acrylic resin (AR) under different applied load

S. no.	Sample code of the composites	Particles used as filler	Optimum concentration of the reinforced filler particles (wt%)	Vickers hardness (HV)		Increase in Vickers hardness (times)	
				Applied load		Applied load	
				2 N	8 N	2 N	8 N
1	AR (acrylic resin)	–	–	28.54	50.73	–	–
2	WC	WHA- as-synthesized whiskers Fig. 1A	0.6	293.34	323.85	10	6
3	CC	CHA- as-synthesized cubes Fig. 1B	0.4	349.78	458.52	12	9
4	C_m_C	Commercial HA Fig. 1C	0.8	133.09	186.66	4.6	3.6

**Table 8:** Comparison of the hardness of the as-synthesized composites (AR, C_m_C, WC, and CC) with the literature

Blank (matrix)	Load (N)	Composite (matrix + fillers)	Fillers	Filler optimum concentration (Wt%) and conditions	Hardness (HV)	References
					Human teeth (Ref.) 350–509 HV	
PMMA	2	AR	–	–	29.3	Present work
		C_m_C	Commercial HA	0.8	130	
		WC	Whiskers HA	0.6	**295**	
		CC	Cubic HA	0.4	**352**	
PMMA	8	AR	–	–	53.1	Present work
		C_m_C	Commercial HA	0.8	187	
		WC	Whiskers HA	0.6	**324**	
		CC	Cubic HA	0.4	**459**	
CP (calcium phosphate)	1.96	CP	–	–	530	[64]
		2% Sr-CP	Sr	2%	531.3	
		4% Sr-CP	Sr	4%	553.7	
		8% Sr-CP	Sr	8%	558	
PVP	1.96	HA-PVP	Rod-shape HA particles	Sintered at 1230 °C	433	[62]
CTAB	1.96	HA-CTAB	Rod-shape HA particles	Sintered at 1200 °C	560	[62]
PMMA (artificial teeth)	2	–	–	Cured for 3 cycles at 30 °C, 95 °C, 155 °C	29.3	[60]
	10	–	–	Cured for 3 cycles at 30 °C, 95 °C, 155 °C	54.3	
HA ceramics	4.9	Rod-shape HA ceramic	–	3 g/ml ceramic sintered at 1250 °C	258	[63]

Bold entries represent optimum properties

Keeping in view the hardness of the teeth that ranges from 305 to 509 HV [63], as well as the hardness of both the as-prepared hybrid composites, it can be suggested that the hybrid composites (WHA–AR and CHA–AR) fabricated in the present study have a potential application in the synthesis of artificial teeth.

## Conclusions

In the present study, the synthesis of HA uniform particles and their reinforcement in the dental AR has been carried out. In addition, the tribological and mechanical behavior of HA-modified composites has been investigated. The conclusions drawn from this study are:

1. Synthesis through homogenous precipitation under controlled experimental parameters resulted in monodispersed fine particle systems of calcium hydroxyapatite.
2. The polymerization technique, along with ultra-sonication, ensured uniform dispersion and a significant interaction of HA within the matrix, thus providing maximum exposure of HA particles when fashioned into the composites for evaluating the tribological and mechanical properties.
3. Wear and frictional behavior were dependent on the interaction between the added nanoparticles and the environment.
4. The evaluation of wear and COF revealed that concentration and morphology played a crucial role in enhancing the tribological properties by forming the tribofilm layer through the exfoliation mechanism.
5. The optimum concentration of the filler particles in the composite was dependent upon the morphological features of the particles.
6. Improvement in the tribological properties of HA particle-reinforced AR composites followed the trend as AR < C_m_C < WC < CC.
7. The as-fabricated composite (CC) having 0.4 Wt% concentration of the cubic-shaped filler particles showed maximum hardness, reduced wear and COF values; thus, it has a potential application in synthesizing artificial teeth.

## Supplementary information

Supplementary information
