# Supplementary figures and images for: Calcium hydroxyapatite nanoparticles as a reinforcement filler in dental resin nanocomposite
Source: J Mater Sci Mater Med. 2021 Oct 3;32(10):129. doi: 10.1007/s10856-021-06599-3 (PMC8487884; doi:10.1007/s10856-021-06599-3)

COVER

**C**


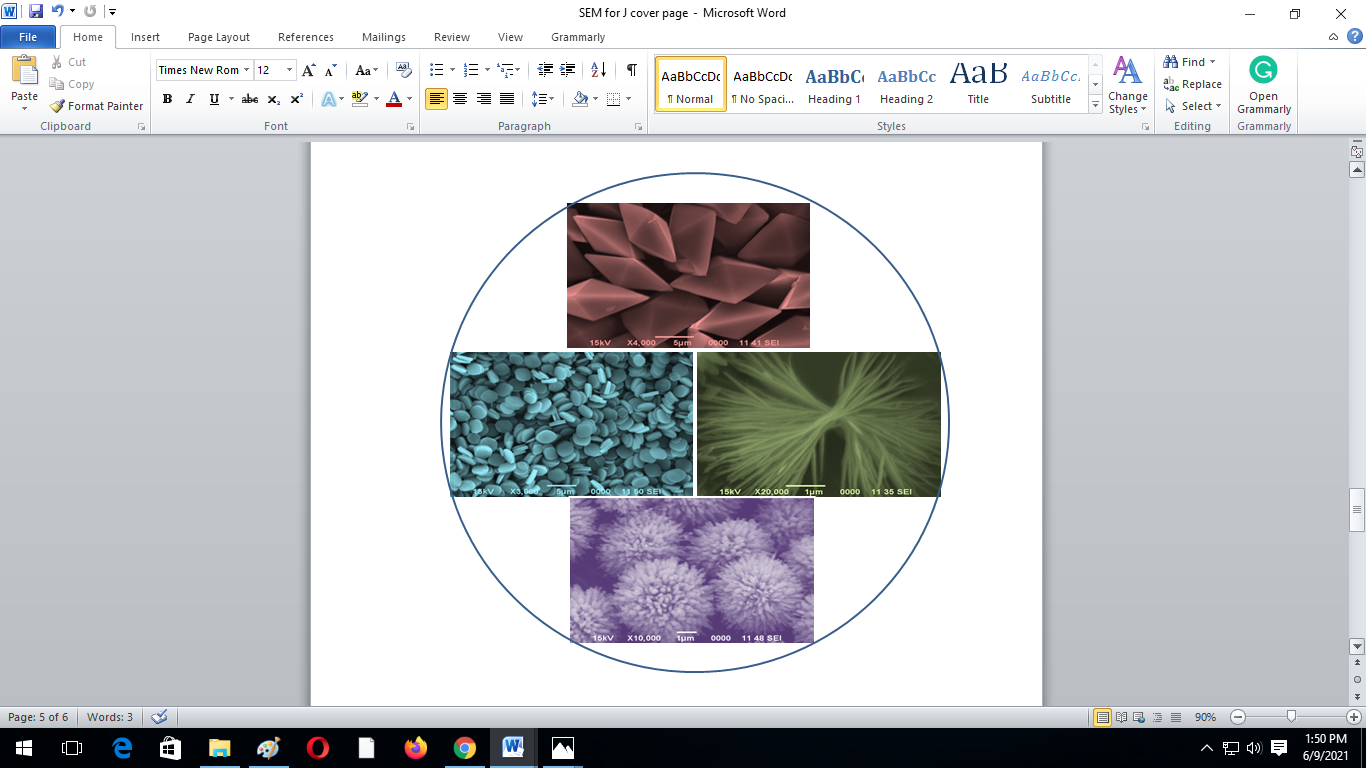

Supplement: Supplementary file 1 — Supplementary information [file 10856_2021_6599_MOESM1_ESM.docx]
